# Supplementary material for: Durable Antimicrobial Behaviour from Silver-Graphene Coated Medical Textile Composites
Source: Polymers (Basel). 2019 Dec 3;11(12):2000. doi: 10.3390/polym11122000 (PMC6961056; doi:10.3390/polym11122000)
Supplement: Supplementary file 1 [file polymers-11-02000-s001.pdf]

Supplementary Information

Durable Antimicrobial Behaviour from Silver-Graphene Coated Medical Textiles Composites

Nuruzzaman Noor [1,*], Suhas Mutalik [1], Muhammad Waseem Younas [1], Cheuk Ying Chan [1], Suman Thakur [1], Faming Wang [1], Mian Zhi Yao [2], Qianqian Mou [2] and Polly Hang-mei Leung [2]

[1] The Hong Kong Polytechnic University, Institute of Textiles and Clothing, Materials Synthesis and Processing Lab, Hung Hom, Kowloon, Hong Kong SAR 999077, China; suhas.mutalik@polyu.edu.hk (S.M.); muhammad.wa.younas@polyu.edu.hk (M.W.Y.); cheuk-ying.chan@connect.polyu.hk (C.Y.C.); suman.thakur@polyu.edu.hk (S.T.); faming.wang@polyu.edu.hk (F.W.)

[2] The Hong Kong Polytechnic University, Department of Health Technology and Informatics, Lee Shau Kee Building, Hung Hom, Kowloon, Hong Kong SAR 999077, China; mian-zhi.yao@connect.polyu.hk (M.Z.Y.); qianqian.mou@connect.polyu.hk (Q.M.); polly.hm.leung@polyu.edu.hk (P.H.L.)

[*] Correspondence: nmnoor@polyu.edu.hk

Results

Zeta potential measurements were used to study the stability and properties of the rGO, Ag and Ag-rGO nanocomposite aqueous precursor mixtures, into which the substrates were immersed (Figure S1).[1–3] $GO_{(aq)}$ had the most negative charge density of −0.89 ± 0.37 mV, and was a highly stable dispersion. The $AgNO_{3(aq)}$ mixture had a slightly more positive, albeit still negative charge density, of -0.76 ± 0.26 mV, still indicating stability, albeit less so than the $GO_{(aq)}$–attributed to the formation of larger agglomerates. Finally, the $GO/AgNO_{3(aq)}$ mixture showed a slightly positive zeta potential value of 0.09 ± 0.12 mV, indicating the lowest stability of all, even though the agglomerations were much smaller. This is because chemically prepared GO sheets tend to possess a multitude of functional groups that can act as repulsive forces when attempting to form multilayered systems; the electrostatic attraction between the anionic GO surface and cationic silver, forms composite that due to the instability, precipitate from solution.[4] This was indeed observed over extended periods of storage for $GO/AgNO_{3(aq)}$ mixtures. However, these observed dispersion effects may be due to the use of water. Other groups have reported variably distributed NP coating samples based on the use of THF, despite both being polar solvents.[5]

After coating, substrates underwent a colour change; due to deposition of AgNPs the colorless substrates turned a yellowish brown; those with rGO turned a grey-black; whilst the Ag-rGO combinations yielded a brown-black colour (Figures S2 and S3). SEM images, bright-field contrast optical microscopic images of fibers as well as longitudinal and cross-sectional images are presented in Figures S4 and S5. The optical microscope images indicate clear variations between the Ag-, rGO-, and Ag-rGO impregnated substrates. The darker coloured aggregates indicate the presence of additives as the result of light absorption or scatter, with the greater density of aggregates in Ag-rGO samples perhaps indicating a higher loading overall.

XRD (Figure S6) confirmed the increasing presence of silver on polyviscose substrates surfaces with increasing $NaBH_{4(aq)}$ concentrations. Comparative particle size trends, using Scherrer's equation on the most intense (111) plane, indicate that there is a larger particle size obtained for Ag-rGO vs. AgNP samples, and increasing $NaBH_{4(aq)}$ yields increasing size for Ag-rGO, whereas the reverse trend is true for the solely AgNP samples (Figure S7a).[6] This was also reflected on the cluster size analysis, as done on *ImageJ*:[7,8] Ag-200 (~170 ± 34nm (before washing) vs. ~194 ± 89nm (after washing)) *vs.* Ag-rGO (~204 ± 57nm (before washing) vs. ~426 ± 180nm (after washing)). Thus, cluster sizes were observed to be larger for Ag-rGO and, after washing, in both cases there was a general cluster size increase although the variation in sizes (i.e.; the degree of disorder), increased significantly, perhaps unsurprisingly.

The slight loading and variation between the different nanocomposite systems is reflected in the calculated fabric thickness values (Figure S7b). Comparing the difference against a blank substrate,

it is clear that any addition of nanocomposite increases the substrate's surface irregularity, however moderately. The largest difference is for AgNP (0.1 gf.cm^{-2}), then rGO (0.09 gf.cm^{-2}) followed by Ag-rGO (0.07 gf.cm^{-2}). These values thus support the data from DLS, SEM and UV-vis; the smaller agglomerates and better distribution in Ag-rGO systems seemingly allowing for a lower substrate thickness increase despite the higher loading detected from EDX.

The experimental UV-vis data for synthesized plasmonic hybrid nanostructured samples demonstrates a well-separated single absorption peak in all cases which corresponds to the surface plasmon resonance (SPR) of spherical AgNP, as encapsulated within the polyviscose fabric substrate (Figure S8).[9,10] SPR red-shifts reflects an increase in particle size, a blue-shift the reverse.[11,12] Narrower SPR bands (i.e.; from the FWHM) is indicative of greater AgNP size distribution uniformity in the sample. Both Ag-rGO and solely AgNP-impregnated substrates display asymmetric peaks at lower $NaBH_{4(aq)}$ concentrations, indicating greater particle size dispersal. This includes some 'tailing' seen at the higher wavelengths, especially for the solely AgNP samples, which indicates that larger fused NP agglomerates are present, as compared to the Ag-rGO, where tailing is heavily diminished, signaling that rGO presence serves to restrict the size of agglomerates.[13] However, there is increasing symmetry where increased $NaBH_{4(aq)}$ is used, corresponding to improved size uniformity as a result of quicker nucleation and faster reduction of Ag^{+} to AgNP.[12] SPR bands show general trends towards progressive intensity increases and hypsochromic peak shifts, as increasing concentrations of $NaBH_{4(aq)}$ are used, indicating the presence of smaller AgNP sizes and agglomerates.[14–16] Generally, at lower concentrations, higher intensities and blue-shifts were observed for Ag-rGO samples, perhaps due to higher loading of metal species. However, by ~200 mmol $NaBH_{4(aq)}$ conditions, SPR band Abs_{max} were approximately equal. This data is also reflected in visible differences between the substrates, proceeding from a pale pink to a golden brown colour for AgNP samples; the Ag-rGO samples tend towards brown-black. In all cases, there is also a clear difference in absorbance envelope trace for the two systems, indicating differing optical responses; the AgNP samples are more responsive to visible light than Ag-rGO systems. Resonance wavelengths strongly depend upon the refractive index of the dielectric medium - it increases if an additional thin layer of high dielectric constant material is over-coated, and decreases for a lower dielectric constant material.[4] Thus, for Ag-rGO, the synergistic plasmonic effects between AgNP and the rGO network, and the differing dielectric constants between the two components, affects the character of the SPR signal.[17] Typically, solely silver presence is said to suffer from chemical instability leading to oxidation that results in SPR red-shift and dampening. However, the inverse relationship was observed here with the involvement of rGO across the series; there was a considerable blue shift of the original signal.

Raman spectra of the impregnated substrates, as well as spectra of the GO and rGO solid precipitates are shown in Figure S9. ATR-FTIR spectra are given in Figure S10. The experimental TGA-DSC data (Figures S11 and S12) supports the findings of low composite loadings relative to the host substrate, with little change in absolute values of thermal transitions for the parent substrate (i.e.; little change in degradation characteristics as a result of Ag-/rGO-/Ag-rGO composites incorporation).[18,19] Broadly, three clear transitions observed that are attributable to the polyviscose; i) ~65 °C due to the loss of bound water; ii) ~345 °C due to degradation of side chain groups of the cellulose fibers with breaking of the main cellulose fibers into small chains, and iii) at ~426 °C due to the complete thermal oxidation of cellulose chains.[20] The only significant change is that the onset of the second transition is raised by ~30 °C in AgNP samples, indicating AgNP presence partially improves the thermal stability of coated fibers.

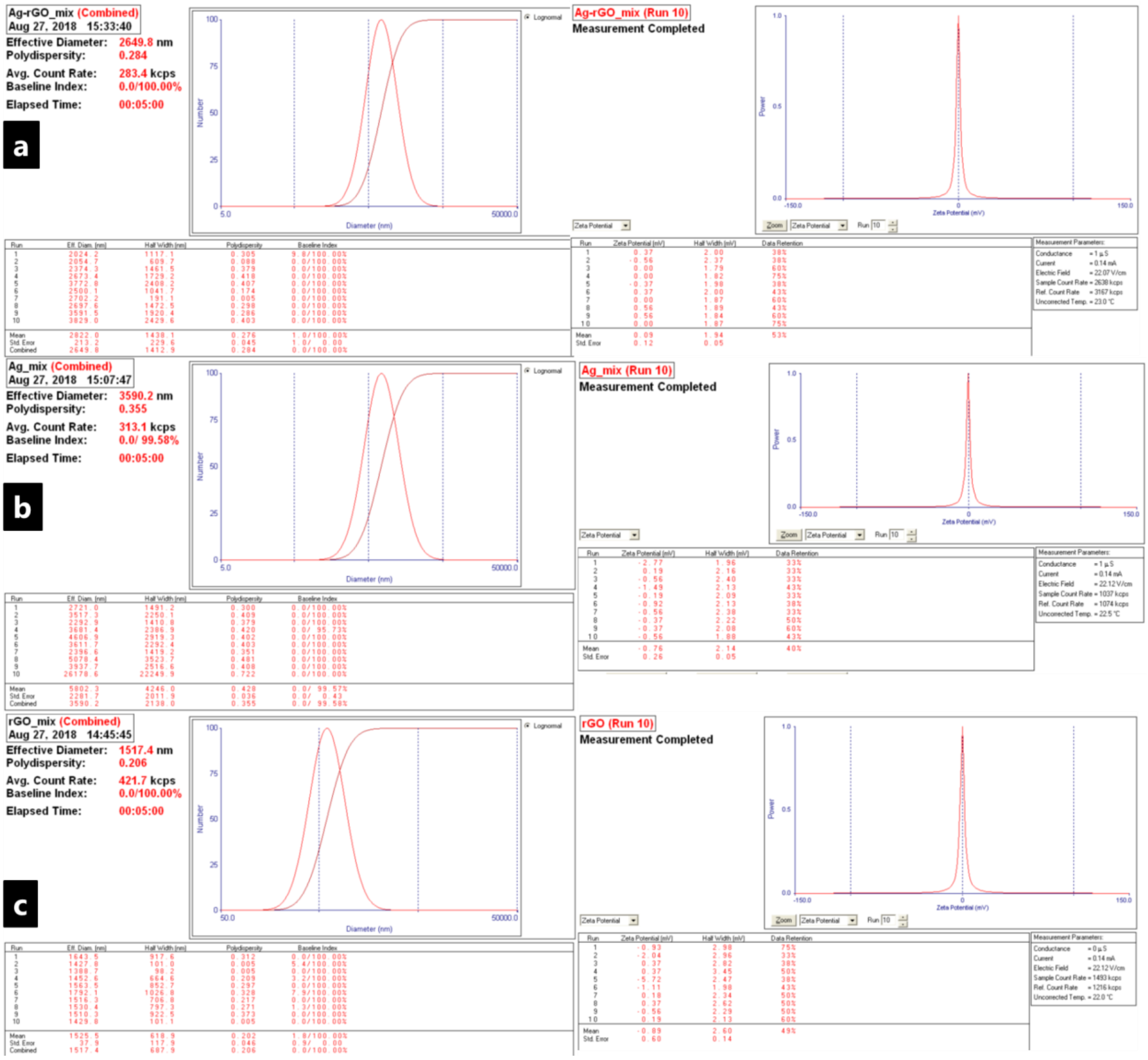

Figure S1. Dynamic light scattering (DLS) and ζ–potential analyses of the; a) silver solution; b) graphene oxide dispersion and; c) silver-graphene oxide mixtures, as used in the experiment for substrate impregnation.

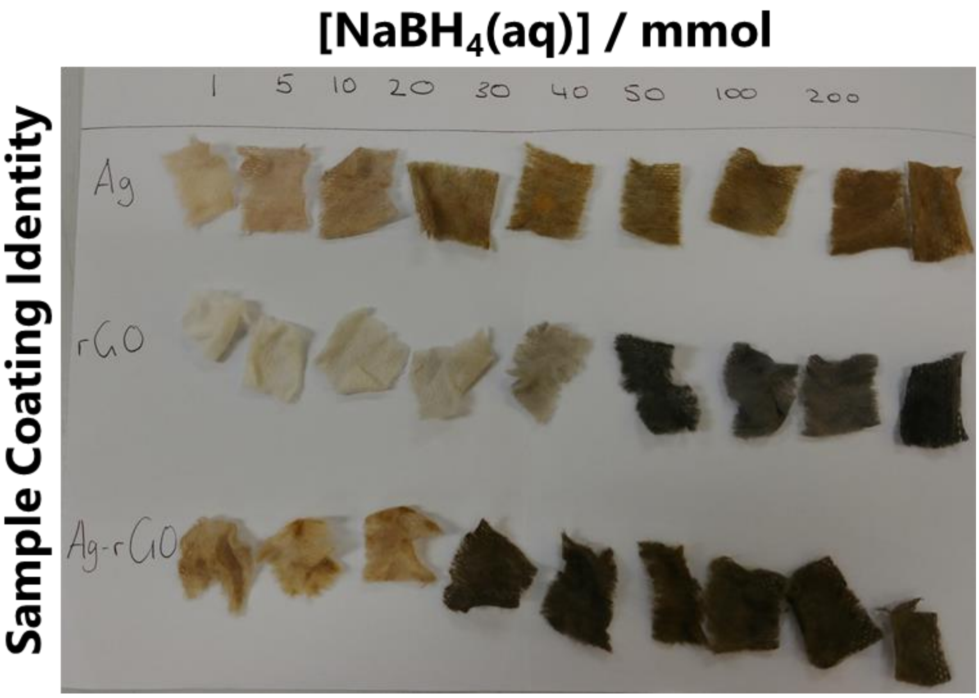

Figure S2. Photograph of sample cuttings comparing active composite loading into polyviscose non-woven fabric substrates, at surface treatment $NaBH_{4(aq)}$ reducing agent concentrations ranging from 1-200 mmol.

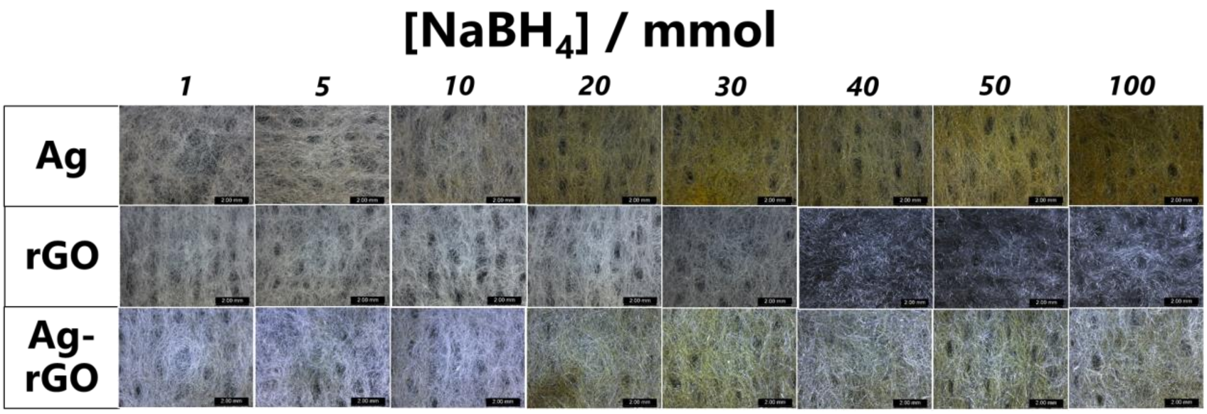

Figure S3. Optical microscope images of Ag/rGO/Ag-rGO active composite loading into polyviscose non-woven fabric substrates, at surface treatment $NaBH_{4(aq)}$ reducing agent concentrations ranging from 1-100 mmol.

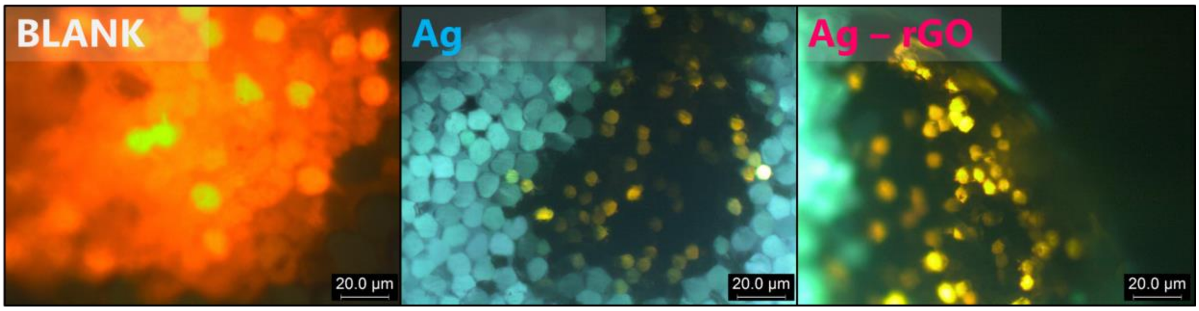

Figure S4. Cross-sectional optical microscope images of blank/Ag/Ag-rGO-incorporated polyviscose fibres, at 200 mmol surface treatment $NaBH_{4(aq)}$ reducing agent concentration indicating the degree of penetration and impregnation.

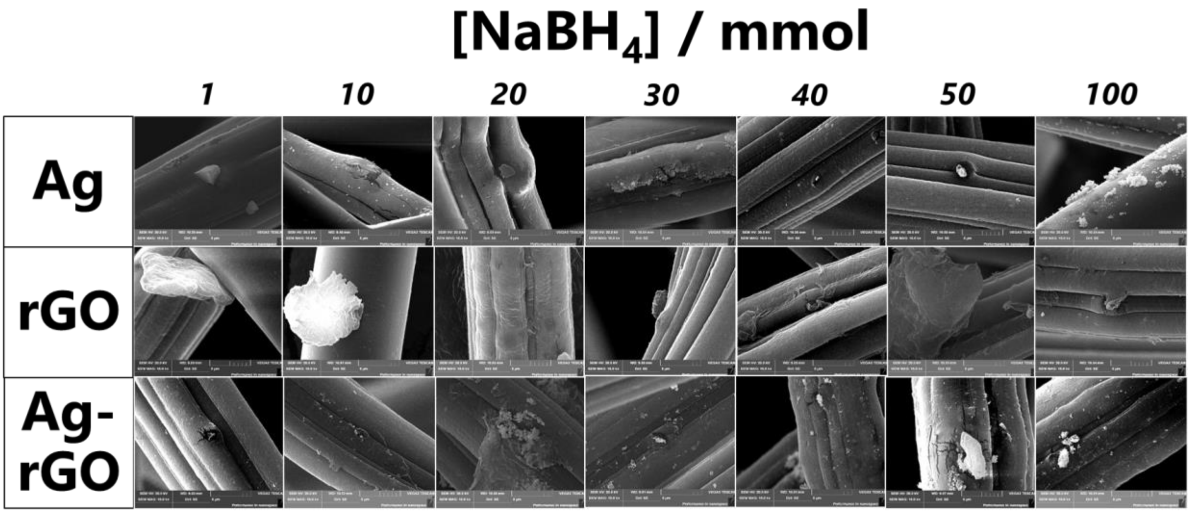

Figure S5. Scanning electron microscopy (SEM) images of AgNP/rGO/Ag-rGO impregnated polyviscose non-woven fabric, at surface treatment $NaBH_{4(aq)}$ reducing agent concentrations ranging from 1-100mmol, confirming the presence of silver and/or reduced graphene oxide lamellar sheets.

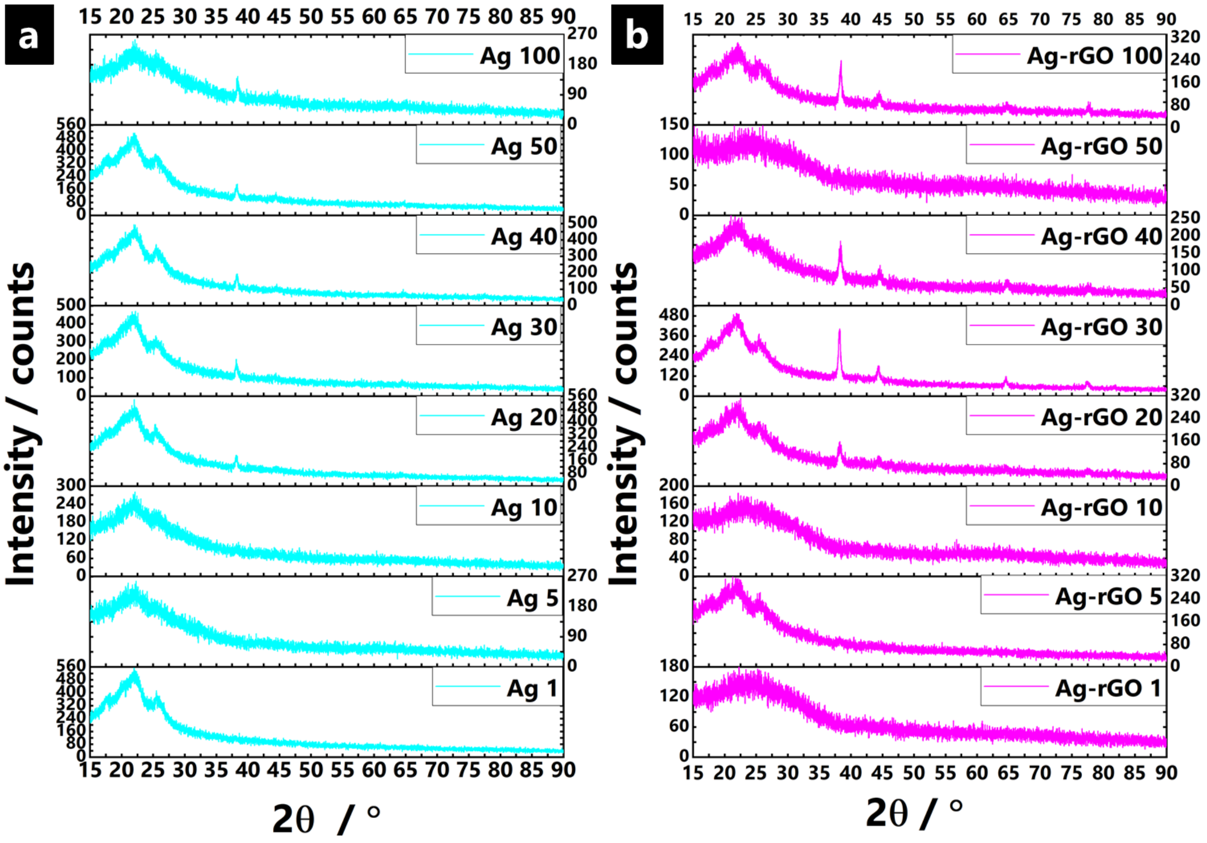

Figure S6. X-ray diffraction (XRD) patterns of a) AgNP, and; b) Ag-rGO-impregnated polyviscose non-woven fabric, at surface treatment $NaBH_{4(aq)}$ reducing agent concentrations ranging from 1-100mmol, confirming the sole presence of fcc metallic silver.

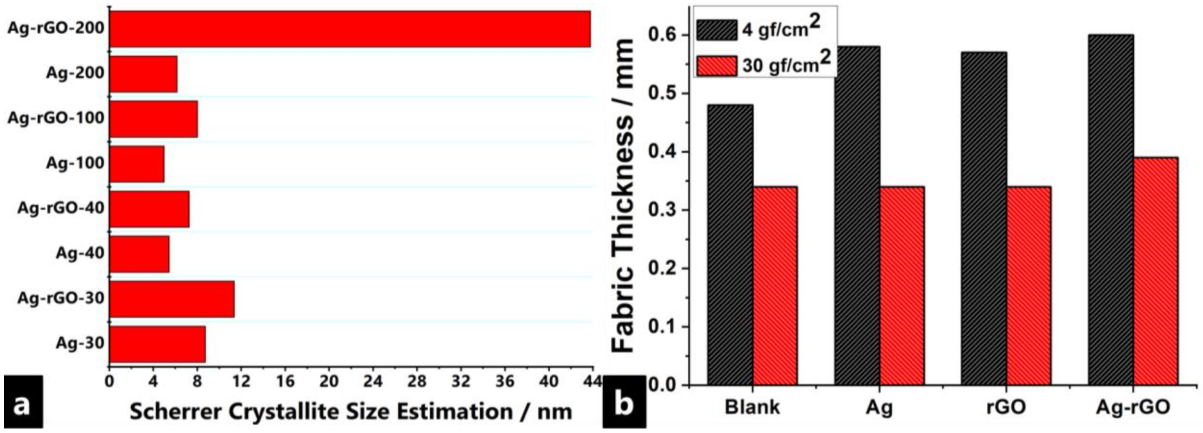

Figure S7. A) Estimated crystallite size data comparisons calculated using the Scherrer method, from fcc metallic silver (111) XRD peaks of i) AgNP, and; ii) Ag-rGO-impregnated polyviscose non-woven fabric, at various surface treatment $NaBH_{4(aq)}$ reducing agent concentrations. B) Fabric thickness test comparisons of AgNP/rGO/Ag-rGO-impregnated polyviscose non-woven fabric, at surface treatment $NaBH_{4(aq)}$ reducing agent concentration of 200mmol.

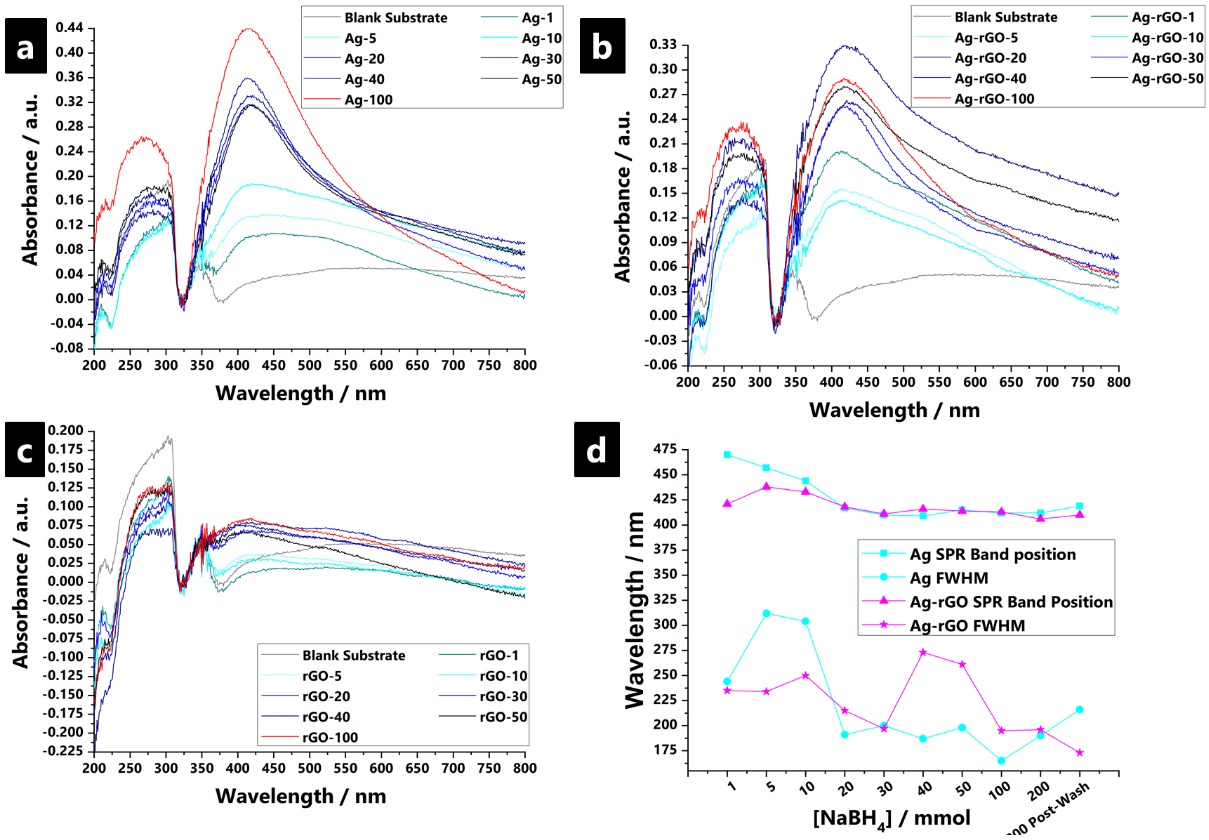

Figure S8. Ultraviolet-visible light (UV-vis) absorption spectra for a) AgNP; b) rGO, and; c) Ag-rGO-impregnated polyviscose non-woven fabric, at surface treatment $NaBH_{4(aq)}$ reducing agent concentrations ranging from 1-100mmol, indicating the surface plasmon resonance (SPR) bands related to silver nanoparticle presence, as well as; d) the SPR band position and FWHM values of the respective composites. *Data for comparison between each series was background subtracted at a single reference wavelength at 320nm for each sample, for ease-of-comparison.

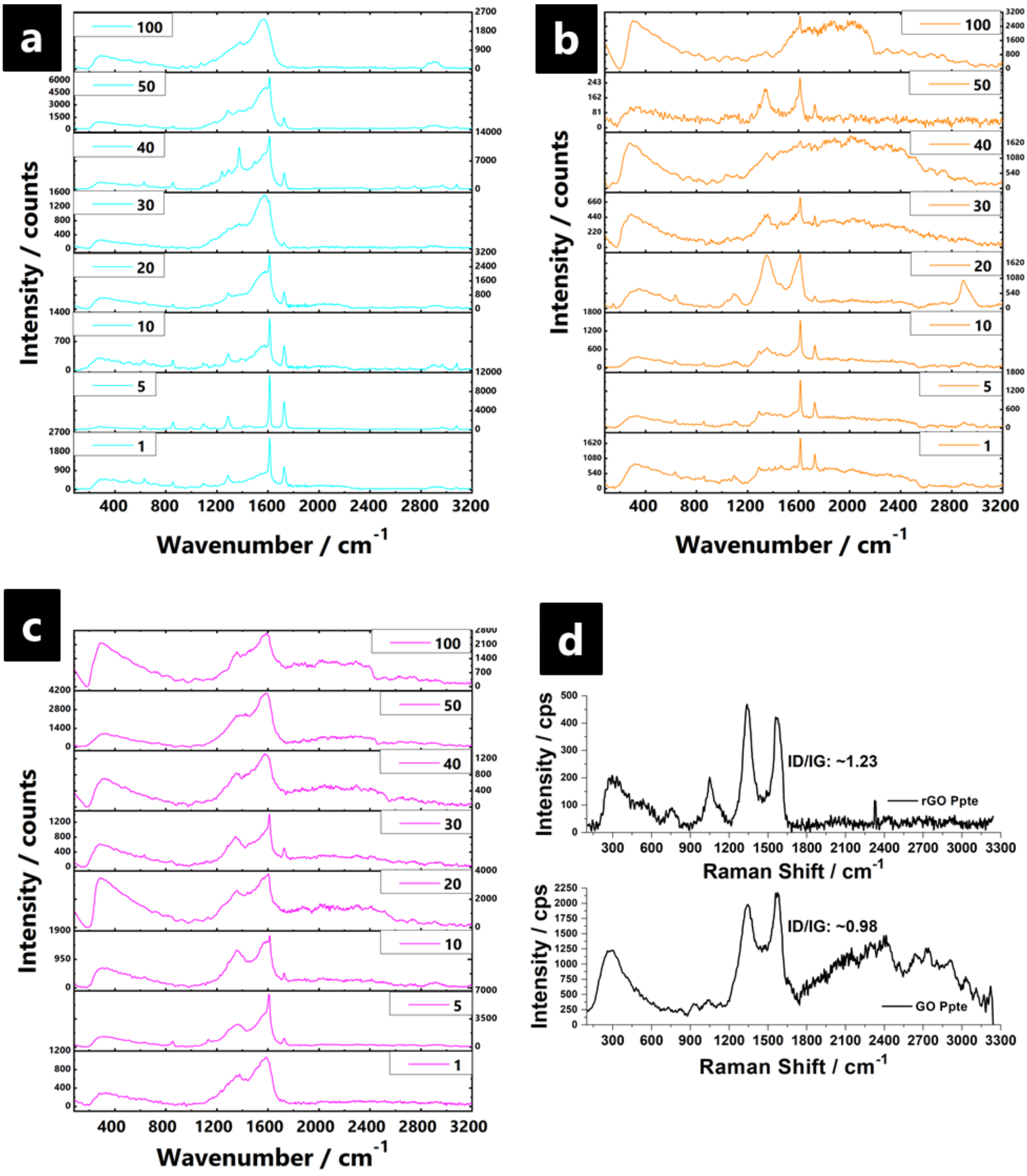

Figure S9. Collated Raman spectra of a) AgNP; b) rGO, and; c) Ag-rGO-impregnated polyviscose non-woven fabric, at surface treatment $NaBH_{4(aq)}$ reducing agent concentrations ranging from 1-100 mmol, acquired under ambient conditions over 100 - 3200 cm^{-1}. The Raman spectra of the dried precipitates of both the graphene oxide (GO) solution starting material, as well as its reduced graphene oxide (rGO) conformer, are given in (d).

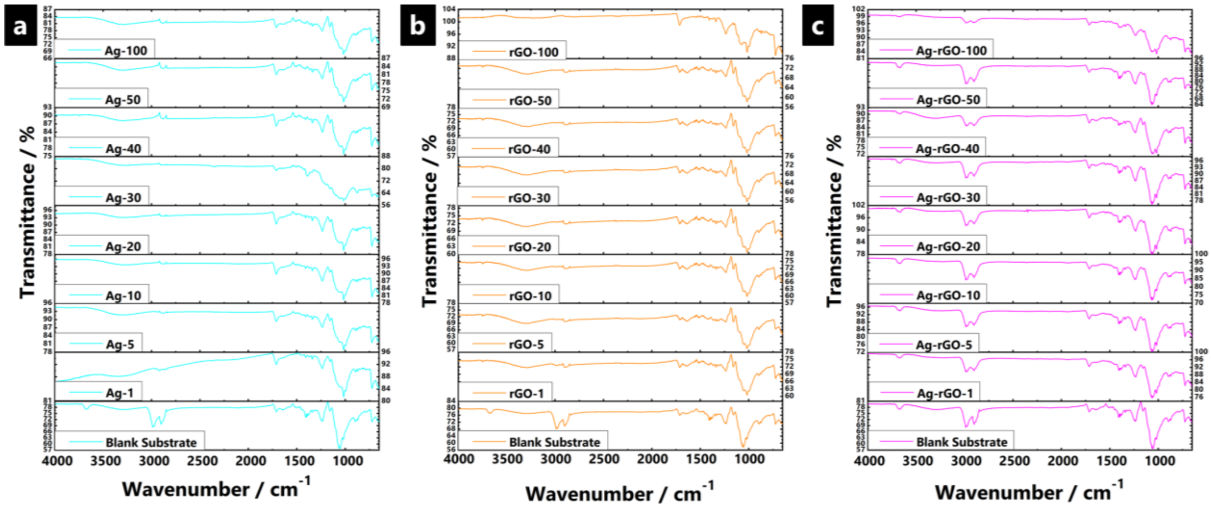

Figure S10. Collated attenuated total reflectance (ATR) - FTIR transmittance spectra of a) AgNP; b) rGO, and; c) Ag-rGO-impregnated polyviscose non-woven fabric, at surface treatment $NaBH_{4(aq)}$ reducing agent concentrations ranging from 1-100mmol, acquired under ambient conditions over 650 - 4000 cm^{-1}.

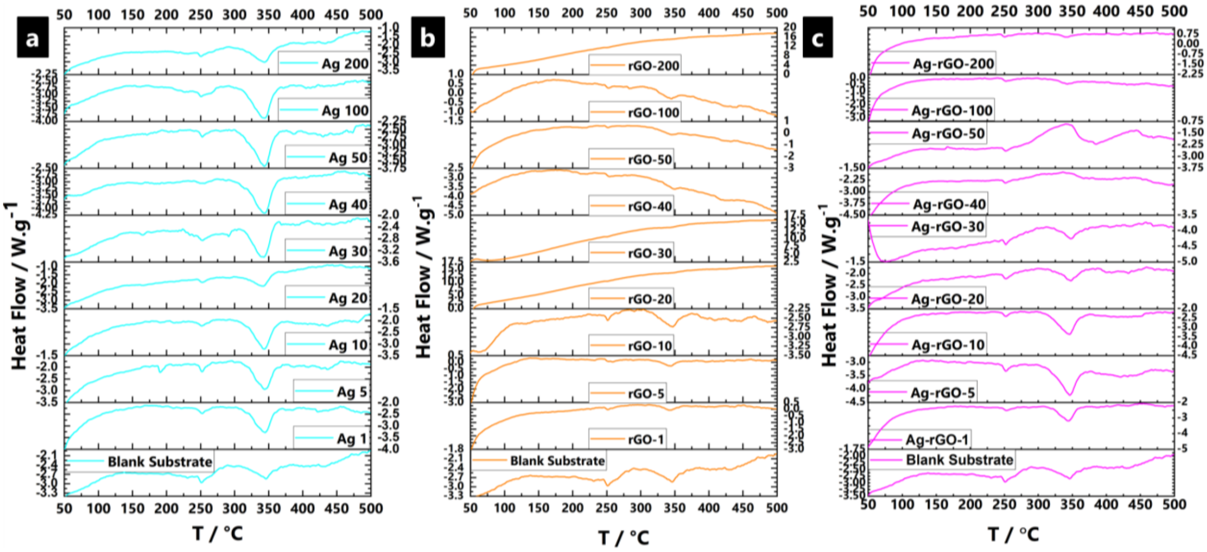

Figure S11. Collated differential scanning calorimetry (DSC) curves of a) AgNP; b) rGO, and; c) Ag-rGO-impregnated polyviscose non-woven fabric, at surface treatment $NaBH_{4(aq)}$ reducing agent concentrations ranging from 1-100mmol, over 50-500°C region.

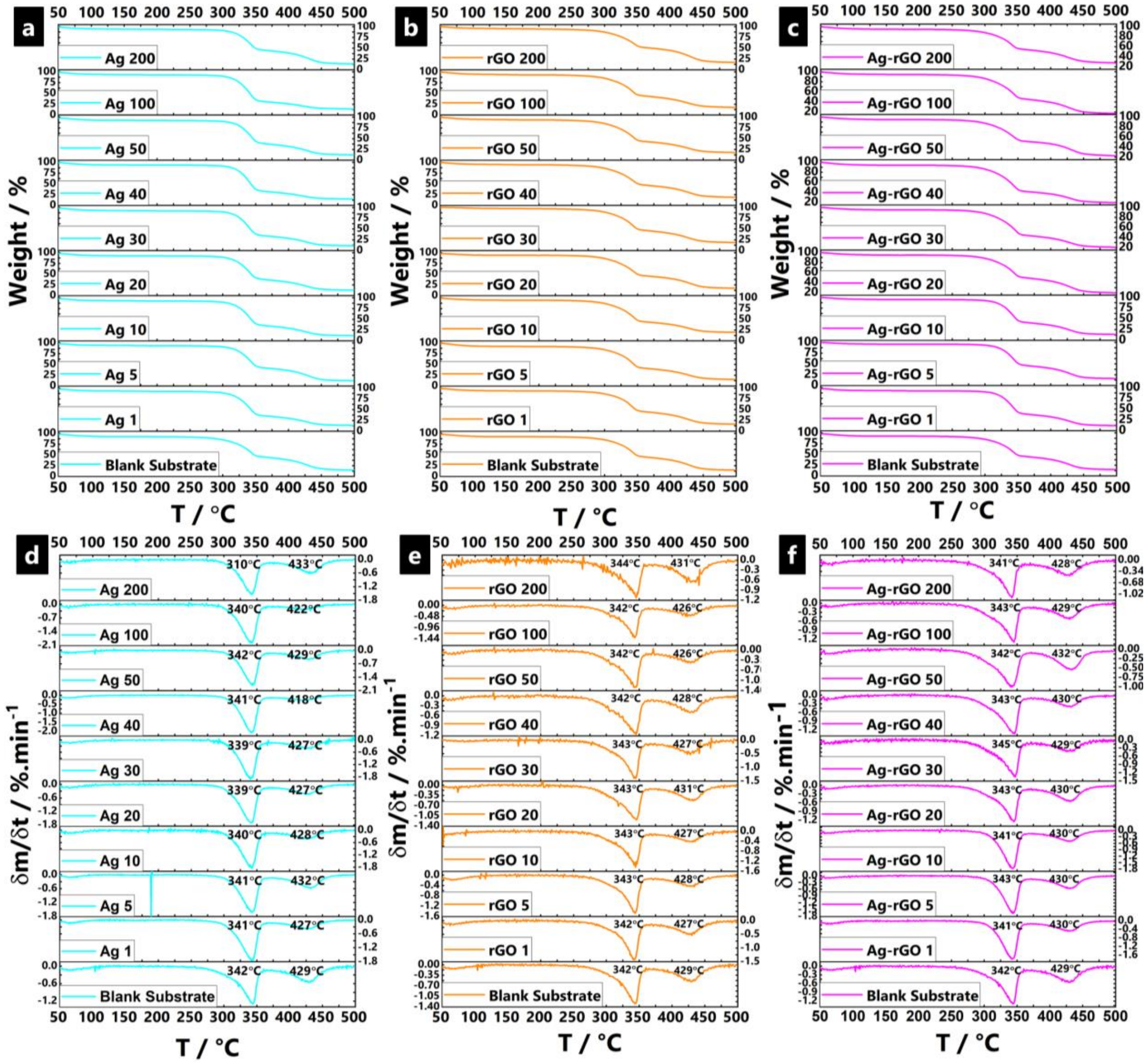

Figure S12. Collated thermogravimetric analyses (TGA) and differential TGA (dTGA) curves respectively of; AgNP (a & d); rGO (b & e), and; Ag-rGO (c & f), impregnated polyviscose non-woven fabric, at surface treatment $NaBH_{4(aq)}$ reducing agent concentrations ranging from 1-200mmol, over 50-500°C region.

References

1. Sakho, E.H.M.; Oluwafemi, O.S.; Saha, A.; Thomas, S.; Kalarikkal, N. Ultrasensitive detection of a 1-pyrenecarboxylic acid by surface enhanced Raman scattering hot spot with reduced graphene oxide/silver nanoparticles composites. *Mater. Lett.* **2016**, *171*, 137–141.
2. Roy, I.; Rana, D.; Sarkar, G.; Bhattacharyya, A.; Saha, N.R.; Mondal, S.; Pattanayak, S.; Chattopadhyay, S.; Chattopadhyay, D. Physical and electrochemical characterization of reduced graphene oxide/silver nanocomposites synthesized by adopting a green approach. *RSC Adv.* **2015**, *5*, 25357–25364.
3. Ali, M.A.; Singh, C.; Srivastava, S.; Admane, P.; Agrawal, V.V.; Sumana, G.; John, R.; Panda, A.; Dong, L.; Malhotra, B.D. Graphene oxide-metal nanocomposites for cancer biomarker detection. *RSC Adv.* **2017**, *7*, 35982–35991.
4. Chung, K.; Rani, A.; Lee, J.E.; Kim, J.E.; Kim, Y.; Yang, H.; Kim, S.O.; Kim, D.; Kim, D.H. Systematic study on the sensitivity enhancement in graphene plasmonic sensors based on layer-by-layer self-assembled graphene oxide multilayers and their reduced analogues. *ACS Appl. Mater. Interfaces* **2015**, *7*, 144–151.
5. Serrano-Montes, A.B.; De Aberasturi, D.J.; Langer, J.; Giner-Casares, J.J.; Scarabelli, L.; Herrero, A.; Liz-Marzán, L.M. A General Method for Solvent Exchange of Plasmonic Nanoparticles and Self-Assembly into SERS-Active Monolayers. *Langmuir* **2015**, *31*, 9205–9213.

6. Muniz, F.T.L.; Miranda, M.A.R.; Morilla dos Santos, C.; Sasaki, J.M. The Scherrer equation and the dynamical theory of X-ray diffraction. *Acta Crystallogr. Sect. A Found. Adv.* **2016**, *72*, 385–390.
7. Rueden, C.T.; Schindelin, J.; Hiner, M.C.; DeZonia, B.E.; Walter, A.E.; Arena, E.T.; Eliceiri, K.W. ImageJ2: ImageJ for the next generation of scientific image data. *BMC Bioinformatics* **2017**, *18*, 529.
8. WURST, J.C.; NELSON, J.A. Lineal Intercept Technique for Measuring Grain Size in Two-Phase Polycrystalline Ceramics. *J. Am. Ceram. Soc.* **1972**, *55*, 109–109.
9. Rivero, P.J.; Urrutia, A.; Goicoechea, J.; Arregui, F.J. Nanomaterials for Functional Textiles and Fibers. *Nanoscale Res. Lett.* **2015**, *10*, 501.
10. Albers, R.F.; Bini, R.A.; Souza, J.B.; Machado, D.T.; Varanda, L.C. A general one-pot synthetic strategy to reduced graphene oxide (rGO) and rGO-nanoparticle hybrid materials. *Carbon N. Y.* **2019**, *143*, 73–84.
11. Gopinath, P.M.; Narchonai, G.; Dhanasekaran, D.; Ranjani, A.; Thajuddin, N. Mycosynthesis, characterization & antibacterial properties of AgNPs against multidrug resistant (MDR) bacterial pathogens of female infertility cases. *Asian J. Pharm. Sci.* **2015**, *10*, 138–145.
12. Maneerung, T.; Tokura, S.; Rujiravanit, R. Impregnation of silver nanoparticles into bacterial cellulose for antimicrobial wound dressing. *Carbohydr. Polym.* **2008**, *72*, 43–51.
13. Tang, B.; Xu, S.; Jian, X.; Tao, J.; Xu, W. Real-Time, in-situ, Extinction Spectroscopy Studies on Silver-Nanoseed Formation. *Appl. Spectrosc.* **2010**, *64*, 1407–1415.
14. Papoff, F.; Hourahine, B. Geometrical Mie theory for resonances in nanoparticles of any shape. *Opt. Express* **2011**, *19*, 21432.
15. Fan, X.; Zheng, W.; Singh, D.J. Light scattering and surface plasmons on small spherical particles. *Light Sci. Appl.* **2014**, *3*, e179–e179.
16. Liu, W.; Oulton, R.F.; Kivshar, Y.S. Geometric interpretations for resonances of plasmonic nanoparticles. *Sci. Rep.* **2015**, *5*, 12148.
17. Genslein, C.; Hausler, P.; Kirchner, E.M.; Bierl, R.; Baeumner, A.J.; Hirsch, T. Graphene-enhanced plasmonic nanohole arrays for environmental sensing in aqueous samples. *Beilstein J. Nanotechnol.* **2016**, *7*, 1564–1573.
18. Duarah, R.; Karak, N. High performing smart hyperbranched polyurethane nanocomposites with efficient self-healing, self-cleaning and photocatalytic attributes. *New J. Chem.* **2018**, *42*, 2167–2179.
19. Layek, R.K.; Nandi, A.K. A review on synthesis and properties of polymer functionalized graphene. *Polymer (Guildf).* **2013**, *54*, 5087–5103.
20. Rehan, M.; Mowafi, S.; Abdelmoez Aly, S.; Elshemy, N.S.; Haggag, K. Microwave-heating for in-situ Ag NPs preparation into viscose fibers. *Eur. Polym. J.* **2017**, *86*, 68–84.

•
